# Sex Attracts: Investigating Individual Differences in Attentional Bias to Sexual Stimuli

**DOI:** 10.1371/journal.pone.0107795

**Published:** 2014-09-19

**Authors:** Sabine Kagerer, Sina Wehrum, Tim Klucken, Bertram Walter, Dieter Vaitl, Rudolf Stark

**Affiliations:** 1 Department of Psychotherapy and Systems Neuroscience, Justus Liebig University Giessen, Giessen, Germany; 2 Bender Institute of Neuroimaging, Justus Liebig University Giessen, Giessen, Germany; Knox College, United States of America

## Abstract

We investigated the impact of sexual stimuli and the influence of sexual motivation on the performance in a dot-probe task and a line-orientation task in a large sample of males and females. All pictures (neutral, erotic) were rated on the dimensions of valence, arousal, disgust, and sexual arousal. Additionally, questionnaires measuring sexual interest/desire/motivation were employed. The ratings of the sexual stimuli point to a successful picture selection because sexual arousal did not differ between the sexes. The stimuli were equally arousing for men and women. Higher scores in the employed questionnaires measuring sexual interest/desire/motivation led to higher sexual arousal ratings of the sex pictures. Attentional bias towards sex pictures was observed in both experimental tasks. The attentional biases measured by the dot-probe and the line-orientation task were moderately intercorrelated suggesting attentional bias as a possible marker for a sex-attention trait. Finally, only the sexual sensation seeking score correlated with the attentional biases of the two tasks. Future research is needed to increase the predictive power of these indirect measures of sexual interest.

## Introduction

There are vast individual differences in the responses to sexual stimuli (e.g. pictures, videos, imagery, fantasies, and touch). These responses can be assessed on a subjective level using questionnaires and on a physiological level using peripheral physiological measures (e.g., genital reactions) or neural responses. Yet, each of these measures has its limitations. Subjective responses - most frequently used - can be influenced by social desirability and cultural standards, which can lead to answer distortions. Peripheral physiological measures have a rather low specificity because they reflect mainly general arousal. Physiological measures such as penis-plethysmography are physically intrusive. Neural measures are hampered by the fact that the functional meaning of the observed brain responses is unclear and findings are still partly inconclusive (cf. [1], [2]).

A further rather different approach for measuring responding to sexual stimuli is the investigation of an attentional bias towards sexual stimuli. Attentional bias refers to the tendency for some stimuli to be preferentially processed, therefore capturing attention. An attentional bias for specific stimuli can be measured by the following procedure: The performance in a cognitive task is compared under two different conditions. In one condition, the stimuli of interest are presented while subjects perform the cognitive task. In the second condition, these stimuli of interest are replaced by neutral stimuli. If subjects are distracted by the stimuli of interest, task performance changes (e.g. faster or slower response times) due to limited attention resources [3]. Thus, attentional bias is reflected in the effects that specific stimuli have on a primary cognitive foreground task.

The effects of emotional stimuli on attention processes have been widely researched in healthy subjects as well as subjects with clinical, mostly anxiety, disorders (cf. [4] for a review, [3] for a meta-analysis). Attentional bias, which might be based on early (preconscious) processing, could provide additional information to subjective questionnaire data, which are based on later, more controlled (conscious) processes. With respect to the processing of sexual stimuli, De Jong [5] in his review suggests that employing approaches that capitalize on attention processes could advance the treatment of sexual dysfunction. Hence, it seems valid to further the investigation of cognitive attention tasks with sexual distractors in order to provide evidence for their applicability.

Evolutionary models of emotion and attention postulate that the visual-attention system is biologically programmed to attend with increased priority to stimuli of biological significance (e.g., [6], [7], [8]), e.g. leading to faster responses in attention paradigms towards relevant stimuli. These can be threat stimuli or stimuli with a positive reward value (primary and secondary reinforces) such as sexual stimuli. Similar to the survival-facilitating mechanism postulated for threat stimuli [9], [10], attentional mechanisms are proposed to aid the rapid detection of sexual cues to ensure reproduction. Consequently, the processing of sexual stimuli should be similar to that of e.g. facial expressions of relevance, automatic and not needing conscious awareness (e.g., [11]). In line with this are the findings by Both and colleagues, who found reflexes to heighten with sexual material [12] and motor readiness to increase with the intensity of the sexual stimuli ([13]). Thus, faster responses towards sex pictures could be expected. In the dot-probe paradigm ([14]), the speeding up in performance on trials in which the target probe appears at the location of the threat-related stimulus might result either from faster engagement with the relevant, important stimulus or from a difficulty to disengage from it (cf. [3]).

Surprisingly, concerning the effects of sexual stimuli, only little research on attentional bias using cognitive tasks has been done. Schimmack and colleagues [15], investigating the effects of emotional stimuli (including sexual stimuli) on reaction times employing two different cognitive tasks (a line detection task and a mathematical problem) found an association between highly arousing sexual pictures and attentional interference. This attention capturing effect of sexually arousing stimuli appeared in some studies even more robust than those caused by negative stimuli [16]. Other studies also found that erotic stimuli attracted more attention than negative stimuli [17], [18].

A further important research question is the association between sexual desire (and related variables) and attentional bias. If a correlation between attentional bias and sexual desire or sexual motivation exists, cognitive reaction time tasks could prove to be an additional useful approach to measure early sexual responding. In this context, Wright and Adams [19] investigated the effects of sexually explicit and neutral stimuli on a choice reaction time with interference task in homosexual and heterosexual males and females. They reported longer reaction times when viewing pictures of potential sexual partners (‘preferred sex slides’); all in all, they conclude that sexual arousal interferes with cognitive processing. In a follow-up study [20], they found similar results. Stimuli which elicited the greatest degree of sexual interest led to the highest interference in the employed discrimination task. Their data indicate that the level of interference changes with the level of sexual arousal elicited by a stimulus. Assuming that individuals with higher sexual desire attend more and respond with more pleasant emotions to sexual stimuli than those with lower levels of sexual desire (based on information processing models (e.g. [21]), Prause and colleagues [22] investigated the responses of healthy participants to sexual stimuli with a dot probe task and a startle eyeblink modulation task. They found that participants with high levels of sexual desire were slower to detect targets in the dot probe task that replaced sexual images, but no such difference was found for the eye-blink task. They propose that the faster responses in the dot detection task of participants with lower sexual desire might have been due to novelty effects. The slower responses of the high sexual desire participants were thought to be due to habituation effects, i.e. due to more previous exposure toward sexual stimuli. In a further study, Brauer et al. [23] employing a single target Implicit Association Task and a dot detection task (similar to that used by Prause et al. [22]) found that reactions in the dot detection task did not differ between healthy women and women with hypoactive sexual desire (despite the fact that the women with hypoactive sexual desire displayed less positive automatic associations with sexual stimuli). Their findings point out that it is important to distinguish between attentional versus affective processes when investigating sexual responding. Moreover, effects of testosterone on attention have been found in males [24] and females with low sexual desire but not in healthy females using oral contraceptives [24]. Despite some inconsistencies in these findings, it has been proposed that the amount of attention captured by sexual stimuli might provide valuable additional information on the behavioural level to the construct of sexual desire/sexual motivation, which is commonly measured by questionnaires [22].

All in all, only few studies have investigated attentional bias to sexual stimuli and even fewer have at the same time looked at sex differences, levels of sexual motivation or sexual arousal. The aim of the present study was therefore to investigate the impact of sexual stimuli and the influence of sexual motivation on the performance in two different attention paradigms in one large sample of males and females: a dot-probe task (cf. [25]; [26]) and a line-orientation task (similar to [27]). The logic of the line-orientation task is the same as in the emotional Stroop task: When the position of the lines has to be indicated, the responses are expected to be slower due to interference of an erotic picture. The dot-detection task allows for the assessment of the direction of attention, with speeded detection times indicating an attentional bias toward the according stimulus category (i.e. sexual stimuli, fear stimuli; [14]). We used two different tasks to explore whether individuals have a stable response pattern, i.e. show similar responses in both tasks.

Subjective sexual motivation/interest was captured with common questionnaires used to measure sexual interest: The Sexual Desire Inventory (SDI, [28]), the Sexual Sensation Seeking Scale (SSSS, [29]) based on the sensation seeking concept of Zuckerman [30], and the Sexual Compulsivity Scale (SCS) by Kalichman [29] based on the concept of sexual compulsivity defined “as an insistent, repetitive, intrusive, and unwanted urge to perform specific acts” [29].

We investigated the following questions (within these questions, we were interested in potentially existing sex differences):

1. Do the two experimental tasks reveal an attentional bias to sexual stimuli?
2. Is task performance influenced by sexual interest/motivation/desire and sexual arousal (as indicated by the ratings of the stimulus material)?
3. Is performance or attentional bias in one task mirrored in the other, i.e. are the measures of interest correlated?

Regarding these questions, we hypothesized that

1. sexual stimuli would lead to an attentional bias (cf. [15], [16], [31], [17], [18]) and that this attentional bias would be more pronounced in males given the stronger sexual motivation in men (cf. [32], [33]) and the previously observed sex differences in the responding to sexual stimuli (cf. [34], [35], [16]);
2. the attentional bias towards sexual stimuli would be influenced by sexual motivation in males and females; the exact influence remains unclear as to the previously inconclusive findings [22]; [23];
3. the attentional biases of the two tasks are correlated, i.e. that there is a stable response pattern.

## Materials and Methods

### Subjects

102 subjects (51 females and 51 males) participated in two attention paradigms. Data of 87 right-handed heterosexual participants (41 females, 46 males) with a mean age of 24.23 (SD = 3.39) were used for statistical analyses. 15 participants were excluded because of negative attitude towards pornography (11 participants), homosexual orientation measured by a German version of the Sexual Orientation Questionnaire (2 participants; SOQ, [36]), medical treatment (1 participant), or missing data (1 participant). Negative attitude towards pornography was assessed by asking each participant: ‘What is your attitude towards pornography?’; the answers possibilities were positive, negative, or neutral. Participants with a negative attitude towards pornography were excluded from analyses because we were interested in the effects caused by sexual arousal, not in the effects of disgust or negatively valenced arousal. Most of the participants were students. No subject was taking medication influencing sexual desire or attention; none of the participants had a history of psychiatric or neurological illness. Volunteers received either course credits or 5 €/h for participation.

### Ethics Statement

Participants were informed about the procedure in detail and gave written informed consent. It was made clear that ‘explicit pornographic material’ would be shown. All experimental procedures were in accordance with the Declaration of Helsinki and were approved by the ethics committee of the German Psychological Society.

### Questionnaires

Participants filled in various questionnaires. The questionnaires relevant for the present study were: Questionnaire on sociodemographic characteristics, Sexual Orientation Questionnaire (SOQ, [36]), Sexual Desire Inventory (SDI, [28]), Sexual Compulsivity Scale (SCS; [29]), and Sexual Sensation Seeking Scale (SSSS; [30]).

### Stimulus material

Sexual stimuli were collected by the authors from the internet and selected in a stepwise procedure. The selection included several steps in order to obtain pictures that were equally sexually arousing for men and women. First, 1,000 pictures were collected by one female and one male investigator, with 500 pictures explicitly depicting genitals (‘hardcore’) and 500 pictures not depicting genitals (‘softcore’). Subsequently, 10 men and 10 women selected the 150 most sexually arousing ‘hardcore’ and the 150 most sexually arousing ‘softcore’ pictures. The selectors were set in a quiet room to look at the pictures on a computer screen. They were instructed to view all pictures and to move the 150 most highly sexually arousing hardcore and the 150 most highly sexually arousing softcore pictures into a specific folder on the PC [37]. This resulted in a selection of 300 sexually arousing pictures. In step 2, these pictures were rated by 100 volunteers (50 male, 50 female) on the dimensions of valence, arousal, sexual arousal, and disgust (valence and arousal with the Self-Assessment-Manikin, [38], sexual arousal and disgust on a 9-point Likert scale). The 72 stimuli (40 dot probe task, 32 line-orientation task) selected for this study had to be rated high in sexual arousal by males and females. Half of the sexual stimuli contained heterosexual hardcore sex pictures with explicit depictions of genitals and oral, vaginal, or anal intercourse (no fetish); the other half were heterosexual softcore pictures, which were less explicit (no depiction of genitals); all sexual stimuli showed scenes with couples (always one man and one woman). The 52 neutral images also collected from the internet depicted men and women in non-sexual interactions. Stimuli were presented on an 18-inch computer screen (1024×768 pixel resolution) using Presentation 12.1. The distance to the screen was 90 cm for all participants. A CRT monitor with 76 Hz and a two-button response pad were used. Pictures were presented in color. At the end of the experimental session, participants rated all pictures on the dimensions of valence and arousal using the Self Assessment Manikin (SAM; [38]), as well as sexual arousal and disgust on a 9-point Likert scale. As reported in the results section, sexual arousal ratings did not differ between hardcore and softcore stimuli; therefore, we decided to combine these two categories into one single category. Please keep in mind that in the following description of the tasks, the sex stimuli always contained half hardcore and half softcore pictures. Consequently, the number of trials including sex pictures doubles the number of trials including neutral pictures.

### Procedure

The study was conducted in one session lasting approximately 2 hours (including training of the attention paradigms and filling in questionnaires). Participants were given specific instructions for each paradigm prior to its performance and were allowed to practice the required responding in order to get accustomed to task and keypad. The paradigms were randomized for order. Between experiments, participants had a short break of approximately five minutes. Responses were given via a two-button keypad. After the experimental tasks, all pictures used in the experiments were rated separately for each picture category on the four rating dimensions (valence, arousal, disgust, and sexual arousal).

#### Dot-probe task

**Figure 1:**
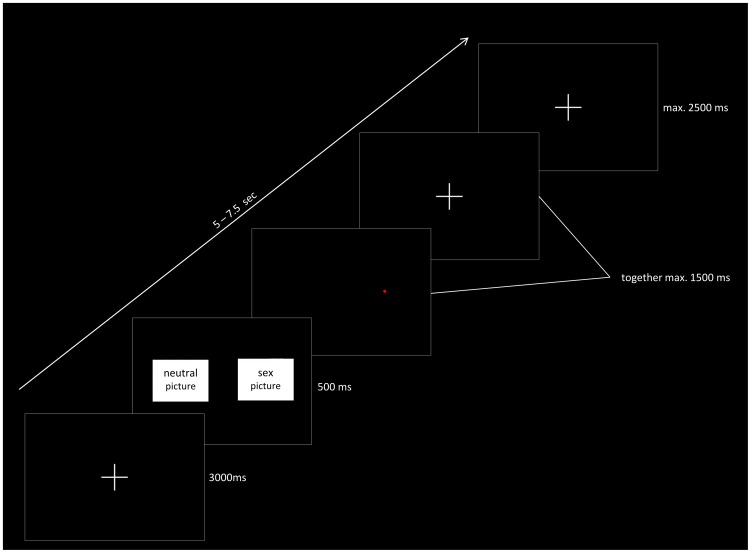
Schematic depiction of a dot-probe trial.

20 neutral pictures and 40 erotic pictures with a 360×270 pixel resolution were shown on a black background (1024×768 pixel). Before each stimulus presentation, a fixation cross (white cross (48×49 pixel) on black background) was shown. Thereafter, two pictures appeared on the left and the right half of the screen; each stimulus pair was presented for 500 ms. Then, a red dot appeared either on the right or the left of the screen central to where one of the pictures had been presented (see Figure 1). Participants had to respond to this dot by pressing the corresponding button (left or right) on the keypad. The maximum presentation time of the dot and thus the maximum response time was 1500 ms. After each response, the fixation cross appeared again and was shown until trial length was 5 s. The fixation cross remained for a variable time window of 0 to 2500 ms (stimulus-onset asynchrony). Pairings consisted of a sex and a neutral picture, two neutral pictures, or two sex pictures. The experiment consisted of 280 trials/picture pairings: 40 neutral-neutral, 160 sex-neutral, and 80 sex-sex picture pairings. Because the categories hardcore and softcore were merged into one, the sex category contained twice as many pictures as the neutral category. The dot was equally often shown on the left or the right side of the screen. In addition, the presentation of the picture categories was controlled for location. This led to the following four experimental conditions: Neutral_Neutral/Neutral_, Sex_Sex/Sex_, Neutral_Sex/Neutral_, Sex_Sex/Neutral_. For example, Sex_Sex/Neutral_ means that the dot appears after a sex/neutral picture pairing at the location of the sex picture. The entire experiment with 280 trials (each 5–7.5 s) lasted for approximately 21 minutes; a different randomization was presented for each participant.

#### Line-orientation task (similar to [27])

32 erotic pictures and 32 neutral pictures with a 480×360 pixel resolution were presented on a black background (1024×768). To the right and to the left of the pictures, short light grey lines were presented. These were tilted by 20°. In half of the cases, the line orientation was the same (parallel, both tilted 20° to the same side); in the other half, the two lines had a different orientation (tilted 20° in opposite directions). The distance between the lines was 548 pixels. Each of the 64 pictures was presented with all four line-conditions. This resulted in altogether 64×4 = 256 different trials. The two different picture categories (sex, neutral) and the two different line orientations (parallel, different) led to the following conditions: sex-parallel, sex-different, neutral-parallel, neutral-different. During the experiment, the subjects had to perform two different tasks: a picture categorization and line-orientation. With the picture categorization instruction, they simply had to indicate as fast as possible with the two-button keypad whether the picture had a sexual or a neutral content. In this task, they had to ignore the lines. With the line-orientation instruction, they had to indicate as fast as possible whether the lines were parallel or different. Thereby, they had to ignore the picture content. Button allocation (left, right) to the two responses within the two tasks was randomized between participants and did not change throughout the experiment.

**Figure 2:**
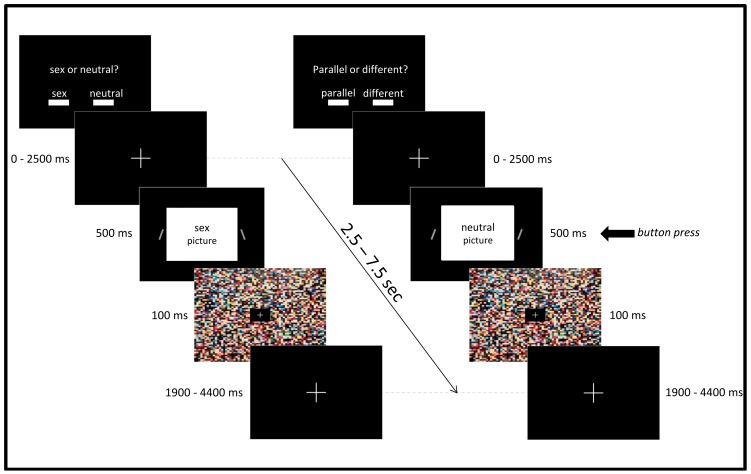
Schematic depiction of the line-orientation task.

The entire experiment consisted of eight task blocks, four picture categorization blocks and four line-orientation blocks. Each block consisted of 32 picture-line-combinations (half erotic and half neutral stimuli, half parallel and half different line orientation). Within a block, no more than 4 sexual or neutral pictures and no more than 5 identical line-orientations were allowed to follow each other. A block started with an instruction (‘sex or neutral?’ or ‘parallel or different?’) on the screen for 5 s. Each trial started with a fixation cross with a variable duration of 0 to 2500 ms. Stimuli were presented for 500 ms; afterwards, a mask with colorful dots was shown for 100 ms to avoid after-images. In order to ensure a trial length of 5 s, the mask was followed by a fixation cross, which was shown for 1900 ms to 4400 ms (see Figure 2).

The dependent variables in this experiment were the reaction times for the four conditions: Sex_Line-orientation_ (line-orientation task, sexual picture as a distractor), Neutral_Line-orientation_ (line-orientation task, neutral picture as a distractor), Sex_Picture categorization_ (picture categorization task, sex picture to categorize), Neutral_Picture categorization_ (picture categorization task, neutral picture to categorize).

The experiment lasted for approximately 22 minutes; a different randomization was presented for each participant.

### Data analyses

Data analyses were performed using SPSS (SPSS Inc., Chicago, Il, USA; Version 19.0). Effect sizes were reported as r or Hedges's g and calculated with confidence intervals (CIs) by the package bootES [39] running under R 3.1.0 [40] using 20.000 resamples. This package computes bias-corrected and accelerated bootstrap CIs as recommended by Kelley [41] for general use.

Mean scores of the questionnaires were compared between the sexes using confidence intervals. Means of the picture ratings were analyzed with mixed factors ANOVAs with Greenhouse-Geisser correction and post hoc pairwise CIs of differences between picture categories. Following Shaffer [42], α = .05 per comparison controls for α = .05 per family of comparisons when the comparison was employed after a significant F-Test of a factor of three levels. For the data analyses of the reaction times (RTs), incorrect responses (cf. [23], [43]) as well as unrealistically fast answers of below 120 ms (cf. [43]) were discarded from the analyses.

#### Dot-probe task

Starting point of the data analyses were the RTs of the four conditions (Neutral_Neutral/Neutral_, Sex_Sex/Sex_, Neutral_Sex/Neutral_, Sex_Sex/Neutral_). Based on these RTs, the scores for *attentional bias, orienting, and disengaging* were calculated. In addition, a *sex activating* score was calculated. The *attentional bias* score (orienting toward and/or disengaging from sex pictures) is based on the RT differences Neutral_Sex/Neutral_ minus Sex_Sex/Neutral_. In consequence, a positive value of the attentional bias score means that the RTs on trials, in which the dot appears at the location of the sex picture of a Sex/Neutral pairing, is faster than in trials, in which the dot appears at the location of the neutral picture of a Sex/Neutral pairing. In order to disentangle the underlying processes of the *attentional bias*, two additional difference scores were calculated: The *orienting* score is based on the RT difference Neutral_Neutral/Neutral_ trials minus Sex_Sex/Neutral_ trials. If this score is positive, the RTs towards dots appearing at the location of a sex picture were faster than RTs towards dots at the location of a neutral picture. The *disengaging* score in contrast is based on RT differences Neutral_Sex/Neutral_ trials minus Neutral_Neutral/Neutral_ trials. If this score is positive, response times in Neutral/Neutral picture pairings were faster than in Neutral/Sex picture pairings. *Sex activating* is based on the RT difference Neutral_Neutral/Neutral_ trials minus Sex_Sex/Sex_ trials. Positive values indicate that the response times are faster after Sex/Sex pairings than after Neutral/Neutral pairings indicating a general facilitation effect of sex pictures independent of the dot location.

*Attentional bias*, *orienting*, *disengaging*, and *sex activating* scores were used for further analyses. For these scores, CIs were used to test whether the scores were different from zero. Further, CIs were calculated to identify sex differences.

#### Line-orientation task

Two relevant difference scores were calculated: (1) *Line-orientation score*: RT in the line-orientation task with sex pictures minus RT with neutral pictures (Sex_Line-orientation_ - Neutral_Line-orientation_). A positive *line-orientation score* indicates that sex pictures prolonged the reaction times by distracting attention from the line-orientation task despite the fact that the picture content was irrelevant. Thereby, participants have to concentrate on a cognitive task while being distracted by a sex picture, i.e. it measures ‘distractibility’ by sex pictures. (2) *Picture categorization score*: RT in the picture categorization task with sex pictures minus RT with neutral pictures (Sex_Picture categorization_ - Neutral_Picture categorization_). A negative *picture categorization score* indicates that sex pictures are categorized faster than neutral pictures. Here, the line orientation was of no interest.

These two RT scores were used for further analysis. CIs were used to test whether the scores were different from zero and CIs were calculated to identify sex differences.

#### Correlation analyses

First, correlations between the questionnaire data and the sexual ratings were conducted. Further, correlations between the questionnaires and the relevant experimental scores of the two experiments were calculated. Finally, correlations within the experimental scores were calculated. Here, the correlations between the experimental scores of the two different experiments are of particular interest. These were calculated for the whole group and separately for males and females. CIs for correlations and for sex differences in correlations were tested by bootstrapping using bootES [39].

For all analyses, α was set to .05 (if not stated otherwise), which leads to 95% CIs. Familywise error control for the tests of the hypotheses specified above was obtained according to the sequential method of Holm [44]. For the decisions on our hypotheses, only tests including all subjects were used.

## Results

### Questionnaire scores

Males and females differ with regard to most of the questionnaire data with males showing higher sexual desire, sexual compulsiveness, and sexual sensation seeking. Only for the SDI solitary scores, no significant sex differences could be shown (see Table 1).

**Table 1:** Means and SD of the SDI and its subscales (Sexual Desire Inventory; rating scale ranging from 0 to 8), the SCS (Sexual Compulsivity Scale; rating scale ranging from 0 to 3), and the SSSS (Sexual Sensation Seeking Scale; rating scale ranging from 0 to 3) for males and females.

	female	male	Hedges's g [95% CI]
SDI total	4.05 (1.32)	4.73 (1.18)	.54 [.09, 1.00]
SDI solitary	3.18 (2.20)	3.71 (1.71)	.27 [−.16, .70]
SDI dyadic	4.91 (0.89)	5.74 (1.11)	.82 [.30, 1.31]
SCS	0.48 (0.40)	0.87 (0.54)	.82 [.37, 1.25]
SSSS	1.49 (0.46)	1.87 (0.39)	.89 [.44, 1.32]

Effect sizes for sex differences are provided with their 95% confidence interval.

All scales of the questionnaires correlate significantly with each other (see Table 2). The high correlation between SDI total and the SDI subscales is trivial and only reported for completeness. Sex differences were found only in the correlation between SCS and SSSS: Males showed a higher correlation than females.

**Table 2:** Intercorrelations of the questionnaire scores; the Sexual Desire Inventory (SDI) with its two subscales, the Sexual Compulsivity Scale (SCS), and the Sexual Sensation Seeking Scale (SSSS) with 95% confidence intervals in brackets.

		SDI solitary	SDI dyadic	SCS	SSSS
**SDI**	all	.92 [.89; .95]	.71 [.60; .79]	.51 [.37; .63]	.44 [.26; .58]
	female	.95 [.91; .97]	.63 [.38; .79]	.51 [.26; .72]	.23 [−.08; .48]
	male	.90 [.83; .94]	.75 [.56; .85]	.44 [.19; .61]	.54 [.31; .69]
**SDI solitary**	all		.39 [.21; .53]	.37 [.20; .51]	.35 [.16; .51]
	female		.36 [.02; .60]	.44 [.15; .65]	.24 [−.09; .49]
	male		.39 [.15; .61]	.29 [.00; .50]	.44 [.21; .63]
**SDI dyadic**	all			.55 [.40; .67]	.41 [.18; .59]
	female			.43 [.14; .68]	.09 [−.30; .46]
	male			.50 [.29; .65]	.49 [.22; .71]
**SCS**	all				.47 [.28; .61]
	female				.17 [−.13; .44]*
	male				.53 [.28; .70]*

*significant sex difference in correlation.

### Picture Ratings

**Figure 3:**
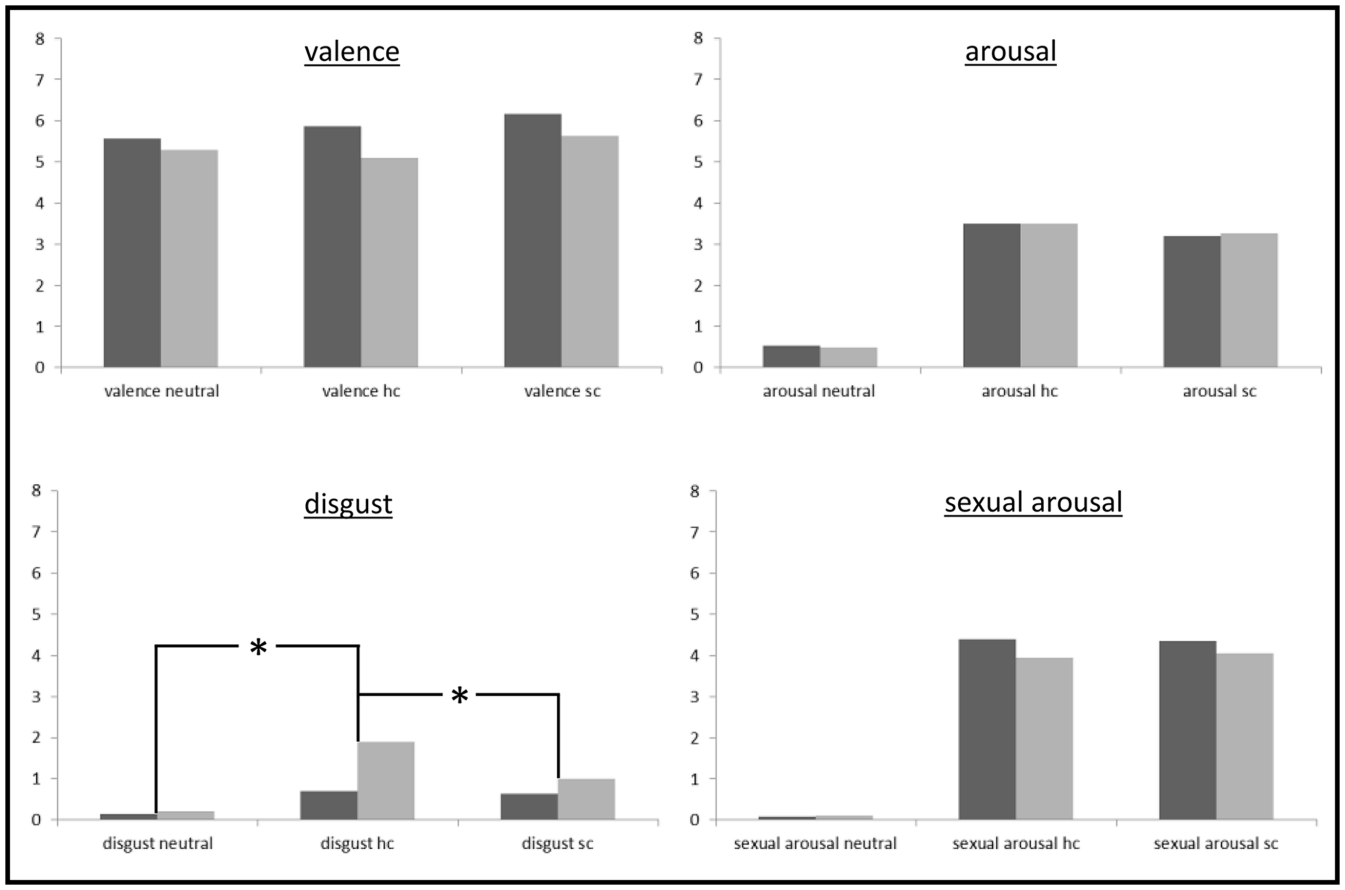
Ratings (disgust, valence, arousal, sexual arousal) of the different picture categories (neutral, softcore, hardcore).

For the four rating measures, 3 (picture category)×2 (sex) ANOVAS were conducted. Results and descriptive data can be seen in Table 3. Figure 3 additionally visualizes the ratings.

**Table 3:** A: Mean subjective ratings, SD and 95% confidence intervals for the three picture categories neutral, hardcore, and softcore of male (n = 46) and female (n = 41) participants (Higher values implying higher positive valence (higher pleasantness), arousal, disgust, or sexual arousal; The rating scales ranged from 0 to 8); B: Analyses of variance of subjective ratings.

A.							
		hardcore		softcore		neutral	
Rating		male	female	male	female	male	female
**valence**	Mean (SD)	5.87 (1.82)	5.10 (1.85)	6.17 (1.43)	5.63 (1.76)	5.57 (1.64)	5.29 (1.72)
	95% CI	[5.26, 6.32]	[4.51, 5.61]	[5.72, 6.54]	[5.05, 6.12]	[5.09, 6.04]	[4.76, 5.81]
**arousal**	Mean (SD)	3.50 (2.33)	3.49 (1.85)	3.20 (2.04)	3,27 (1.77)	.52 (.94)	.49 (.75)
	95% CI	[2.83, 4.15]	[2.88, 4.02]	[2.59, 3.78]	[2.71, 3.78]	[.30, .85]	[.29, .73]
**disgust**	Mean (SD)	.70 (1.13)	1.90 (2.07)	.63 (1.12)	1.00 (1.40)	.15 (.36)	.22 (.613)
	95% CI	[.41, 1.07]	[1.32, 2.59]	[.37, 1.05]	[.61, 1.46]	[.04, .26]	[.07, .46]
**sexual arousal**	Mean (SD)	4.39 (2.26)	3.95 (2.26)	4.35 (2.40)	4.05 (1.92)	.09 (.29)	.10 (.37)
	95% CI	[3.72, 5.00]	[3.27, 4.59]	[3.63, 5.00]	[3.42, 4.59]	[.02, .17]	[.00, .24]

B.								
	valence		arousal		disgust		sexual arousal	
Factor	F	p	F	p	F	p	F	p
**Picture category**	3.90	.036	172.41	<.001	28.17	<.001	269.79	<.001
**Sex**	3.16	.079	.00	.977	7.48	.008	.61	.437
**Interaction**	.90	.379	.05	.918	7.88	.001	.64	.480

For valence, arousal, and sexual arousal only main effects of picture category were found. The picture categories differed only marginally with respect to the valence ratings. They were highest for softcore and lowest for neutral pictures (softcore – neutral: D = .48 [.09; .85]; softcore – hardcore: D = .42 [.21; .69]). The difference between hardcore and neutral pictures was not significant (D = .06 [−.42; .47]).

Remarkable differences between picture categories were observed for arousal and especially for sexual arousal. For arousal, the difference between both sex picture sets and the neutral pictures was more than 2 scale units (hardcore – neutral: D = 2.99 [2.60; 3.40]; softcore – neutral: D = 2.73 [2.36; 3.10]) and for sexual arousal more than 4 scale units (hardcore – neutral: D = 4.08 [3.61; 4.52]; softcore – neutral: D = 4.11 [3.64; 4.53]). For arousal, differences between hardcore and softcore pictures were small (hardcore – softcore; arousal: D = .26 [.02; .51]). For sexual arousal, differences between hardcore and softcore pictures were not significant (hardcore – softcore: sexual arousal: D = −.03 [−29; .21]).

Concerning the disgust ratings, all picture categories were rated rather low (all means below .2). Despite this restricted range of the disgust ratings, an interaction of sex and picture category was found. Compared to neutral pictures, hardcore pictures elicited more disgust in females than in males (females: D = 1.68 [1.10; 2.39]; males: D = .54 [.26; .89]; females – males: D = 1.14 [.48; 1.94]). A similar sex specific response was seen when comparing hardcore and softcore pictures (females: D = .90 [.59; 1.29]; males: D = .07 [−.33; .33]; females – males: D = .84 [.40; 1.39]). Both sexes rated also softcore pictures as more disgusting than neutral pictures (females: D = .78 [.37; 1.27]; males: D = .48 [.24; .83]; females – males: D = .30 [−.24; .85]).

### Correlations between sexual arousal and questionnaire scores

All questionnaire scores (SDI, SDI solitary and SDI dyadic, SCS, SSSS) correlated significantly with the sexual arousal ratings of the sex pictures (mean of the hardcore and softcore ratings; see Table 4): The higher the sexual desire, sexual compulsivity, and the sexual sensation seeking, the higher the sexual arousal ratings. No sex differences with regard to these correlation coefficients were found.

**Table 4:** Correlations (with 95% confidence intervals in brackets) between questionnaire scores and sexual arousal for males, females, and the entire group.

		sexual arousal
**SDI**	all	**.42 [.24;.58]**
	female	**.44 [.09; .66]**
	male	**.41 [.14; .61]**
**SDI solitary**	all	**.36 [.16; .51]**
	female	**.38 [.03; .61]**
	male	**.34 [.09; .54]**
**SDI dyadic**	all	**.37 [.19; .52]**
	female	**.37 [.08; .59]**
	male	**.36 [.10; .57]**
**SCS**	all	**.33 [.08; .53]**
	female	.20 [−.33; .49]
	male	**.40 [.07; .63]**
**SSSS**	all	**.32 [.07; .51]**
	female	.20 [−.16; .47]
	male	**.43 [.07; .65]**

Confidence intervals not encompassing zero are written in bold.

### Effects of each of the experiments

#### Dot-probe task

The mean reaction times of the dot-probe task as well as the scores for *attentional bias, orienting, disengaging*, and *sex activating* can be seen in Table 5. Scores for attentional bias and disengaging but not for orienting and sex activating were greater than zero, as indicated by the CIs. The effects of viewing sex picture on attention was rather small (maximum g = .33). No sex differences could be shown in reaction times or scores by bootstrap CIs (all g<.29).

**Table 5:** Dot-probe task: Mean reaction times [ms], SD and 95% confidence intervals (in brackets) for the trial conditions and the four difference scores for males, females, and the entire group.

Trial condition	female (n = 41)	male (n = 46)	all (n = 87)
Neutral_Sex/Neutral_	440.6 (58.9) [423.7; 459.6]	449.5 (74.3) [430.8; 473.4]	445.3 (67.2) [432.5; 460.9]
Sex_Sex/Neutral_	435.1 (56.4) [418.6; 453.0]	435.5 (66.7) [418.8; 457.6]	435.3 (61.7) [423.2; 449.4]
Sex_Sex/sex_	433.9 (56.4) [417.9; 451.9]	440.2 (67.5) [422.8; 463.1]	437.2 (62.3) [425.1; 451.6]
Neutral_Neutral/Neutral_	436.3 (53.6) [420.8; 452.9]	443.3 (69.0) [425.5; 465.9]	440.0 (61.9) [427.8; 453.8]
**Scores**			
Attentional bias Neutral_Sex/Neutral_ – Sex_Sex/Neutral_	5.4 (22.0) [−.6; 12.7]	14.0 (34.6) [6.2; 26.6]	10.0 (29.5)* [4.7; 17.7]
g	.24 [−.05; .52]	.40 [.11; .58]	.33 [.15; .48]
Orienting Neutral_Neutral/Neutral_ – Sex_Sex/Neutral_	1.1 (21.4) [−5.2; 7.7]	7.8 (28.5) [.5; 16.8]	4.7 (25.5) [−.4; 10.2]
g	.05 [−.26; .36]	.27 [−.04; .51]	.18 [−.03; .37]
Disengaging Neutral_Sex/Neutral_ – Neutral_Neutral/Neutral_	4.3 (21.1) [−1.6; 11.2]	6.2 (18.2) [1.5; 11.8]	5.3 (19.6)* [1.4; 9.5]
g	.20 [−.11; .48]	.33 [.04; .59]	.27 [.06; .46]
Sex activating Neutral_Neutral/Neutral_ – Sex_Sex/sex_	2.4 (14.8) [−2.4; 6.6]	3.2 (16.8) [−1.5; 8.2]	2.8 (15.8) [−.5; 6.2]
g	.16 [−.16; .48]	.19 [−.10; .47]	.18 [−.03; .39]

*significant after familywise error correction for the tests of four scores using all subjects.

For difference scores Hedges's g with confidence intervals are added.

#### Line-orientation task

The mean reaction times in the line-orientation task and the respective scores can be seen in Table 6. CIs indicate that the line-orientation score and the picture categorization score were different from zero. Rather large effects (g = .76 and −.62) could be observed. No sex differences could be shown in reaction times or scores by bootstrap CIs (all g<.15).

**Table 6:** Line-orientation task: Mean reaction times [ms], SD and 95% confidence intervals (in brackets) for the trial conditions and the two difference scores for males, females, and the entire group.

Trial condition	female (n = 41)	male (n = 46)	all (n = 87)
Sex_Line-orientation_	786.8 (151.7) [745.0; 838.0]	777.8 (176.8) [732.5; 834.7]	782.0 (164.6) [751.0; 818.9]
Neutral_Line-orientation_	754.9 (135.4) [718.0; 801.0]	751.6 (166.6) [709.2; 804.3]	753.2 (151.8) [724.5; 788.3]
Sex_Picture categorization_	538.9 (135.5) [509.8; 600.6]	536.9 (110.8) [509.3; 574.1]	537.9 (122.3) [516.9; 570.7]
Neutral_Picture categorization_	566.1 (112.5) [539.7; 609.6]	569.5 (108.0) [542.6; 604.8]	567.9 (109.5) [548.1; 594.3]
**Scores**			
line-orientation Sex_Line-orientation –_ Neutral_Line-orientation_	31.8 (41.5) [20.5; 45.8]	26.2 (34.5) [17.0; 37.1]	28.9 (37.9)* [21.2; 37.4]
g	.75 [.43; 1.00]	.75 [.45; 1.03]	.76 [.54; .95]
picture categorization Sex_Picture categorization -_ Neutral_Picture categorization_	−27.2 (55.7) [−43.1; −9.3]	−32.5 (40.5) [−44.8; −21.1]	−30.0 (48.0)* [−39.6; −19.4]
g	−.48 [−.85; −.08]	−.79 [−1.01; −.49]	−.62 [−.87; −.33]

*significant after familywise error correction correction for the tests of two scores using all subjects.

For difference scores Hedges's g with confidence intervals are added.

### Correlations between questionnaires and experimental scores

With regard to the relationship between the questionnaires scores (SDI, and its subscales SDI solitary and SDI dyadic, SCS, and SSSS) and the experimental scores (attentional bias, orienting, disengaging, sexual activating, line-orientation score, picture categorization score), the analysis for the whole group revealed familywise corrected significant correlations between SSSS and the orienting and picture categorization scores (see Table 7). Males and females differed only in the correlations between SDI dyadic and attentional bias as well as SCS and picture categorization (not familywise error corrected).

**Table 7:** Correlations (with 95% confidence intervals in brackets) between questionnaire and task scores for males, females, and the entire group.

		attentional bias	orienting	disengaging	sexually activating	line-orientation	picture categorization
**SDI**	all	.13 [−.05; .28]	.08 [−.09; .24]	.08 [−.12; .26]	.09 [−.11; .29]	−.09 [−.29; .09]	.11 [−.12; .31]
	female	.05 [−.27; .37]	−.05 [−.32; .22]	.10 [−.24; .41]	.09 [−.22; .37]	−.01 [−.28; .23]	.19 [−.17; .47]
	male	.12 [−.08; .32]	.12 [−.07; .33]	.04 [−.19; .27]	.08 [−.19; .34]	−.15 [−.42; .13]	.04 [−.21; .30]
**SDI solitary**	all	.05 [−.11; .19]	.02 [−.14; .17]	.04 [−.15; .24]	.07 [−.14; .27]	−.05 [−.23; .14]	.19 [−.03; .38]
	female	.10 [−.22; .41]	−.03 [−.32; .24]	.14 [−.19; .46]	.09 [−.23; .39]	.06 [−.19; .31]	.23 [−.12; .50]
	male	−.02 [−.21; .13]	.03 [−.13; .21]	−.10 [−.31; .10]	.04 [−.25; .31]	−.18 [−.43; .14]	.15 [−.09; .38]
**SDI dyadic**	all	**.21 [.02; .42]**	.16 [−.04; .39]	.12 [−.09; .33]	.08 [−.11; .30]	−.13 [−.35; .13]	−.08 [−.29; .13]
	female	−.11 [−.36; .17]°	−.06 [−.27; .28]	−.05 [−.34; .23]	.03 [−.24; .35]	−.19 [−.50; .12]	.01 [−.31; .29]
	male	**.30 [.04; .53]** °	.21 [−.06; .53]	.24 [−.03; .47]	.11 [−.16; .39]	−.03 [−.34; .30]	−.14 [−.46; .14]
**SCS**	all	.20 [−.06; .45]	.15 [−.11; .41]	.10 [−.14; .35]	.09 [−.14; .31]	.04 [−.16; .29]	−.01 [−.24; .24]
	female	.20 [−.19; .46]	−.06 [−.41; .24]	.28 [−.08; .66]	−.08 [−.47; .24]	.16 [−.22; .52]	.31 [−.04; .66]°
	male	.13 [−.26; .48]	.20 [−.20; .51]	−.06 [−.35; .33]	.17 [−.13; .45]	.02 [−.25; .36]	−.26 [−.52; .04]°
**SSSS**	all	**.24 [.02; .43]**	**.33 [.13; .50]** *	−.07 [−.25; .15]	**.28 [.09; .45]**	.16 [−.03; .35]	**−.24 [−.39; −.09]** *
	female	−.04 [−.36; .35]	.17 [−.10; .48]	−.21 [−.43; .02]	**.28 [.02; .49]**	**.33 [.13; .49]**	−.19 [−.36; .05]
	male	**.37 [.06; .62]**	**.42 [.12; .68]**	.04 [−.22; .36]	**.32 [.04; .57]**	.05 [−.32; .38]	**−.31 [−.58; −.05]**

° significant sex difference in correlation (no familywise error correction).

*significant after familywise error correction for the tests of 30 correlations (5 questionnaires by 6 experimental scores) using all subjects.

Confidence intervals not encompassing zero are written in bold.

### Correlations between the experimental scores

The correlation coefficients between the experimental scores can be seen in Table 8. Whereas most scores of the dot-probe task are highly correlated, there was no correlation between the two scores delineated from the line-orientation task. Correlations between the two sets of experimental scores were moderate. Only the correlation between orienting and picture categorization remained significant after familywise error correction. Sex differences could only be shown in the correlations within the dot-probe scores: stronger correlations were found in males between attentional bias and orienting and in females between disengaging and sexually activating.

**Table 8:** Intercorrelations (with 95% confidence intervals in brackets) of the experimental scores for the entire group and for males and females separately.

		attentional bias (AB)	orienting (OR)	disengaging (DE)	sexually activating (SA)	line-orientation (LO)	picture categorization (PC)
**AB**	all		**.76 [.55; .87]**	**.52 [.35; .70]**	**.31 [.09; .52]**	.15 [−.04; .32]	−.23 [−.47; .01]
	female		**.53 [.14; .75]** °	**.51 [.24; .70]**	.14 [−.23; .45]	.03 [−.24; .28]	−.10 [−.39; .26]
	male		**.85 [.65; .94]** °	**.57 [.26; .83]**	**.40 [.13; .64]**	.27 [−.02; .48]	−.34 [−.67; .01]
**OR**	all			−.16 [−.48; .27]	**.70 [.50; .80]**	.16 [−.08; .37]	**−.36 [−.54; −.14]** *
	female			−**.47 [**−**.69;** −**.19]**	**.79 [.65; .87]**	.00 [−.31; .30]	**−.44 [−.66; −.13]**
	male			.05 [−.47; .56]	.65 [.35; .80]	.32 [−.06; .58]	−.31 [−.63; .03]
**DE**	all				**−.44 [−.63; −.05]**	.03 [−.22; .25]	.13 [−.20; .50]
	female				**−.66 [−.82; −.38]** °	.03 [−.22; .29]	.33 [−.13; .74]
	male				−.26 [−.57; .31]°	.02 [−.40; .43]	−.16 [−.53; .21]
**SA**	all					.12 [−.12; .33]	**−.29 [−.50; −.05]**
	female					.04 [−.29; .30]	−.27 [−.61; .11]
	male					.20 [−.20; .51]	−.32 [−.60; .02]
**LO**	all						−.14 [−.33; .07]
	female						−.16 [−.43; .17]
	male						−.12 [−.40; .13]

^°^significant sex difference in correlation (no familywise error correction).

*significant after familywise error correction for the tests of 8 correlations (4 dot-probe scores by 2 line-orientation scores) using all subjects.

Confidence intervals not encompassing zero are written in bold.

## Discussion

The aim of the present study was to explore the attentional bias towards sexual stimuli as well as the influence of sex and individual sexual motivation (measured by questionnaires) in a healthy student sample. Two different experimental tasks that measured attentional bias were performed.

### Questionnaires measuring sexual interest/desire/motivation

Regarding the questionnaire data, commonly observed sex differences (cf. [34], [32], [33]) between males and females could be confirmed. We found significantly higher subjective reports of sexual desire, sexual compulsiveness, and sexual sensation seeking in males than in females.

Although the different questionnaire scores correlated significantly with each other, the questionnaires seem to measure different aspects of sexuality (shared variance of approximately 20–25%).

### Ratings of the sexual stimuli

Interestingly, sexual arousal ratings of the softcore and the hardcore pictures were similar and this was the case for males and females. This led us to merge the two picture categories. Regarding sex differences, the hardcore pictures were rated as more disgust-inducing by females than males. All other ratings showed no significant sex differences. This points to a successful selection of pictures because one would expect sex differences in the ratings of sexual material without such a specific selection procedure (cf. [35]; [45]). The similar rating was most likely due to the selection process of the pictures, which were rated beforehand as highly sexually arousing by men and women. This is in line with other studies [46],[47], who found reactivity towards sexually explicit media to depend on the material used.

### Sexual arousal ratings and sexuality related attitudes

A correlation between the questionnaire data and sexual arousal ratings was observed: The higher the sexual desire, sexual compulsivity, sexual sensation seeking, the higher the sexual arousal ratings. This is in line with e.g. Rupp et al. [45] and Brand et al. [48], who suggest that factors such as sexual motivation influence picture ratings and even the tendency to watch pornographic material on the internet.

### Attentional bias - effects of the paradigms

In general, we found pictures with sexual content to influence attention in both paradigms, but the effect sizes were small.

The dot-probe task revealed an *attentional bias* for sex pictures, i.e. participants were faster when they had to respond to sex pictures with Sex/Neutral picture combinations. Looking in more detail at the *attentional bias*, the dot probe score *disengaging* revealed that participants were slowed down or distracted by a sex picture when they had to respond to a neutral picture. Thus, participants had problems disengaging, i.e. directing their attention away from a sex picture. Yet, they did not experience significantly enhanced *orienting*. Therefore, the attentional bias can assumedly be traced back more to problems in disengaging from sexual pictures than to an enhanced orienting towards sexual pictures. Until now, only few studies have used the dot-probe paradigm to measure attentional bias towards sexual stimuli. Interestingly, Prause and colleagues [22] found an attentional bias in the opposite direction in a healthy sample of males and females. Here, the subjects reacted faster when the dot appeared at the position of the neutral picture after a Sex/Neutral picture pair, whereas the participants in the present study responded faster when the dot appeared in the position of the sex picture after a Sex/Neutral picture pair. Brauer et al. [23], who investigated females with hypoactive sexual desire disorder and healthy controls using a shortened version of the paradigm by Prause et al. [22], reported a similar result. The authors tried to explain the prolonged reaction times towards sexual pictures with participants being more absorbed by the sex pictures leading to slower responses. This discrepancy with the findings by Prause et al. (faster responses to neutral pictures) and our findings (faster responses to sex pictures) need to be explained in the future. Unfortunately, Prause et al. [22] do not report their raw reaction times but only z-scores. However, Brauer and colleagues [23] report reaction times much higher than our reaction times (approximately 100–150 ms). This might possibly be due to different methodological or response procedures with Brauer et al. [23] and Prause et al. [22]. Speculatively, differences in the findings could be due to differences in the intensity of the stimulus material, the inclusion of other pleasant and unpleasant stimuli, or different keypad procedures used. Future research needs to elucidate these diverging findings. Generally, attentional bias is expected to lead to faster responses towards sex pictures in the dot-detection task ([14]). Though, other studies using e.g. positive smoking cues, found processing delays rather than a faster processing (e.g., [49]). In our sample, heightened internal motivation due to an increase in arousal might have led to faster motor responses in the dot-detection task (and the picture-categorization task, see below for details). This is in line with Both et al. [12], who found reflexes to heighten with sexual material compared with neutral stimuli. In a further study ([13]), they found motor readiness to increase with the intensity of the sexual stimuli.

Further, no significant sex differences were found regarding the attentional bias score. This is in accordance with Prause et al. [22], who also report no sex-differences in their dot detection task.

Considering the line-orientation task, participants responded altogether faster with picture categorization than with line-orientation. As expected, attentional bias was observed for both relevant difference scores: Regarding the picture categorization score, participants responded faster to the emotionally charged sex pictures than to the neutral pictures. Regarding the line-orientation score, participants responded slower with background sex pictures than with background neutral pictures. Thus, sex pictures made responses faster when the attention was focused on the pictures (cf. [12], [13]); but when line-orientation had to be determined, the sex pictures seem to have captured more attention than the neutral pictures making it harder to focus on the task, which led to slower responses. This resembles the disengaging problem observed in the dot-probe task. Similar to our line-orientation task findings, Stippekohl and colleagues ([43]) found faster responses to smoking cues than to control stimuli in their line-orientation task when the focus was on the picture (picture-categorization score). They also found slower responses in the line-orientation task, i.e. more distraction due to background smoking pictures. In addition, the present findings are in line with Most et al. [16] who found positively arousing stimuli to cause ‘emotion-induced deficits in visual processing’. In their experiments, participants were distracted by the sexual stimuli despite monetary incentives offered for ignoring the distractors. The observation that the categorization of the sex pictures was faster than that of neutral pictures is again in line with previous findings in studies with healthy participants (cf. [12], [13], [50]), in which sexual stimuli led to a heightened readiness for motor responses due to the high arousal elicited by sexual stimuli. Furthermore, for neither of the two attentional bias scores of the line-orientation task, sex differences were observed. Contrary to our findings, in a different kind of line-detection task, Schimmack et al. [15] found that pictures with same-sex models produced more attentional interference for females than for males, whereas opposite sex-models distracted males more than females. The discrepancies to the study by Schimmack et al. [15] might be explained by different stimulus material. The stimulus material used in the present study depicted always sexual scenes with males and females interacting. Also, pictures were selected to minimize sex differences in sexual arousal and to specifically appeal to both sexes.

### Correlations of sexuality-related attitudes and attentional bias

A small individual variability in attention allocation was observed depending on the scores in the questionnaires measuring sexuality-related attitudes. Only for the SSSS, we found higher *attentional bias* (*orienting* and *picture categorization*) scores with higher scores of sexual sensation seeking. Since we investigated a healthy sample, the variance of the questionnaire scores might have been too low to reveal more significant correlations. Other samples need to be investigated in order to clarify, e.g. the influence of sexual compulsivity on attention. We did not observe any important sex differences. The observed correlations are again discrepant to the findings by Prause et al. [22], who reported a negative correlation between sexual desire and attentional bias. But despite this discrepancy, attentional bias towards sexual material seems to be related to sexuality-related attitudes (i.e., sexual sensation seeking).

Attention paradigms could possibly be used as indirect index for sexual attitudes. Keeping in mind that questionnaire data are open to the influence of social desirability and deception, paradigms measuring attentional bias might provide a validation of self-report data in that they measure sex-related attention processes. A more indirect assessment, less affected by social desirability and deception, could be useful. Still, future research needs to examine incremental and divergent validity of self-report vs. indirect approaches in order to prevent prematurely adopting cost-ineffective tools thereby possibly even losing predictive power. The present correlations are rather low and future research is required to increase the predictive power of these indirect measures. Furthermore, correlations might be higher if implicit attitudes were assessed using implicit attitude tests such as the Implicit Association Test ([51]; cf. [52]) or the Single Target Implicit Association Task (cf. [23]) instead of using questionnaires. The assessment of implicit attitudes could be added to further validate findings in the future.

### Intercorrelations of the attention tasks

The only significant correlation between the paradigm scores was observed for the dot-probe orienting score and the picture categorization score of the line-orientation task. This makes sense because both scores measure the amount of attention capturing of sexual stimuli due to speeded up responses. The fact that there is a correlation between the scores of the two paradigms suggests a stable individual response pattern pointing to a more trait-like sex-related attentional bias.

Future research should investigate attentional bias with other tasks. It should be possible to find the task with the highest incremental validity for assessing sexual motives in addition to questionnaires, which are prone to be biased by the self-concept of an individual.

### Limitations

Further research should include other positive stimuli in order to filter out valence effects. The set of pictures used needs to be re-examined possibly omitting stimuli depicting practices which could be negatively evaluated by some subjects (e.g. anal sex). In addition to the long duration of the dot probe task, it has one other disadvantage, i.e. that the lack of the dot in one location points to the presence of the dot in the other location and vice versa. The use of the line-orientation task should thus possibly be favored in future. Last, different presentation times (250 ms vs. 500 ms) could be employed. Shorter presentation times could investigate the influence of inhibition of return ([53]), an orienting phenomenon that slows down visual attention to a previously searched location. Additionally, information on whether participants tend to perform (or avoid) saccades towards specific stimuli could be collected.

## References

[1] Georgiadis J, Kringelbach M (2012) The human sexual response cycle: Brain imaging evidence linking sex to other pleasures. Progress in Neurobiology 98 (1) 49–81. 22609047 10.1016/j.pneurobio.2012.05.004

[2] Georgiadis JR, Reinders AS, Paans AM, Renken R, Kortekaas R (2009) Men versus women on sexual brain function: Prominent differences during tactile genital stimulation, but not during orgasm. Hum Brain Mapp 30 (10) 3089–3101. 19219848 10.1002/hbm.20733 PMC6871190

[3] Bar-Haim Y, Lamy D, Pergamin L, Bakermans-Kranenburg MJ, van IJzendoorn MH (2007) Threat-related attentional bias in anxious and nonanxious individuals: A meta-analytic study. Psychological Bulletin 133 (1) 1–24. 17201568 10.1037/0033-2909.133.1.1

[4] Yiend J (2010) The effects of emotion on attention: A review of attentional processing of emotional information. Cognition & Emotion 24 (1) 3–47.

[5] Jong DC de (2009) The Role of Attention in Sexual Arousal: Implications for Treatment of Sexual Dysfunction. Journal of Sex Research 46 (2–3) 237–248. 19308846 10.1080/00224490902747230

[6] LeDoux JE (1996) The emotional brain. The mysterious underpinnings of emotional life. New York: Simon & Schuster. 384 p.

[7] Panksepp J (1998) Affective neuroscience. The foundations of human and animal emotions. New York: Oxford University Press. xii, 466 p.

[8] Rolls ET (2000) The Orbitofrontal Cortex and Reward. Cerebral Cortex 10 (3) 284–294. 10731223 10.1093/cercor/10.3.284

[9] Mathews A, Mackintosh B (1998) Cognitive Therapy and Research 22 (6) 539–560.

[10] Mogg K, Bradley BP (1998) A cognitive-motivational analysis of anxiety. Behaviour Research and Therapy 36 (9) 809–848. 9701859 10.1016/s0005-7967(98)00063-1

[11] Morris JS, Ohman A, Dolan RJ (1998) Conscious and unconscious emotional learning in the human amygdala. Nature 393 (6684) 467–470. 9624001 10.1038/30976

[12] Both S, Everaerd W, Laan E (2003) Modulation of spinal reflexes by aversive and sexually appetitive stimuli. Psychophysiology 40 (2) 174–183. 12820858 10.1111/1469-8986.00019

[13] Both S, Spiering M, Everaerd W, Laan E (2004) Sexual behavior and responsiveness to sexual stimuli following laboratory-induced sexual arousal. Journal of Sex Research 41 (3) 242–258. 15497053 10.1080/00224490409552232

[14] MacLeod C, Mathews A, Tata P (1986) Attentional bias in emotional disorders. Journal of Abnormal Psychology 95 (1) 15–20. 3700842 10.1037//0021-843x.95.1.15

[15] Schimmack U, Derryberry D (2005) Attentional Interference Effects of Emotional Pictures: Threat, Negativity, or Arousal. Emotion 5 (1) 55–66. 15755219 10.1037/1528-3542.5.1.55

[16] Most SB, Smith SD, Cooter AB, Levy BN, Zald DH (2007) The naked truth: Positive, arousing distractors impair rapid target perception. Cognition & Emotion 21 (5) 964–981.

[17] Anderson AK (2005) Affective influences on the attentional dynamics supporting awareness. J Exp Psychol Gen 134 (2) 258–281. 15869349 10.1037/0096-3445.134.2.258

[18] Buodo G, Sarlo M, Palomba D (2002) Attentional Resources Measured by Reaction Times Highlight Differences Within Pleasant and Unpleasant, High Arousing Stimuli. Motivation and Emotion 26 (2) 123–138.

[19] Wright LW, Adams HE (1994) Assessment of sexual preference using a choice reaction time task. J Psychopathol Behav Assess 16 (3) 221–231.

[20] Wright LW, Adams HE (1999) The effects of stimuli that vary in erotic content on cognitive processes. Journal of Sex Research 36 (2) 145–151.

[21] Barlow DH (1986) Causes of sexual dysfunction: The role of anxiety and cognitive interference. Journal of Consulting and Clinical Psychology 54 (2) 140–148. 3700800 10.1037//0022-006x.54.2.140

[22] Prause N, Janssen E, Hetrick WP (2008) Attention and Emotional Responses to Sexual Stimuli and Their Relationship to Sexual Desire. Arch Sex Behav 37 (6) 934–949. 17943435 10.1007/s10508-007-9236-6

[23] Brauer M, Leeuwen M, Janssen E, Newhouse SK, Heiman JR, et al (2012) Attentional and Affective Processing of Sexual Stimuli in Women with Hypoactive Sexual Desire Disorder. Arch Sex Behav 41 (4) 891–905. 21892693 10.1007/s10508-011-9820-7

[24] Alexander G, Shervin B (1993) Sex steroids, sexual behavior, and selection attention for erotic stimuli in women using oral contraceptives. Psychoneuroendocrinology 18 (2) 91–102. 8493300 10.1016/0306-4530(93)90060-x

[25] Posner MI, Snyder CR, Davidson BJ (1980) Attention and the detection of signals. J Exp Psychol 109 (2) 160–174. 7381367

[26] MacLeod C, Mathews A, Tata P (1986) Attentional bias in emotional disorders. Journal of Abnormal Psychology 95 (1) 15–20. 3700842 10.1037//0021-843x.95.1.15

[27] Pessoa L, Padmala S, Morland T (2005) Fate of unattended fearful faces in the amygdala is determined by both attentional resources and cognitive modulation. NeuroImage 28 (1) 249–255. 15993624 10.1016/j.neuroimage.2005.05.048 PMC2427145

[28] Spector IP, Carey MP, Steinberg L (1996) The sexual desire inventory: Development, factor structure, and evidence of reliability. Journal of Sex & Marital Therapy 22 (3) 175–190. 8880651 10.1080/00926239608414655

[29] Kalichman SC, Rompa D (1995) Sexual Sensation Seeking and Sexual Compulsivity Scales: Validity, and Predicting HIV Risk Behavior. Journal of Personality Assessment 65 (3) 586–601. 8609589 10.1207/s15327752jpa6503_16

[30] Zuckerman M, Kolin EA, Price L, Zoob I (1964) Development of a sensation-seeking scale. Journal of Consulting Psychology 28 (6) 477–482. 14242306 10.1037/h0040995

[31] Most SB, Chun MM, Widders DM, Zald DH (2005) Attentional rubbernecking: cognitive control and personality in emotion-induced blindness. Psychon Bull Rev 12 (4) 654–661. 16447378 10.3758/bf03196754

[32] Baumeister RF, Catanese KR, Vohs KD (2001) Is There a Gender Difference in Strength of Sex Drive? Theoretical Views, Conceptual Distinctions, and a Review of Relevant Evidence. Personality and Social Psychology Review 5 (3) 242–273.

[33] Hiller J (2005) Gender differences in sexual motivation. The Journal of Men's Health & Gender 2 (3) 339–345.

[34] Baumeister RF (2000) Gender differences in erotic plasticity: The female sex drive as socially flexible and responsive. Psychological Bulletin 126 (3) 347–374. 10825779 10.1037/0033-2909.126.3.347

[35] Rupp HA, Wallen K (2007) Sex differences in viewing sexual stimuli: An eye-tracking study in men and women. Hormones and Behavior 51 (4) 524–533. 17362952 10.1016/j.yhbeh.2007.01.008

[36] Sell RL (1996) The Sell Assessment of Sexual Orientation: Background and scoring. Journal of Gay, Lesbian, & Bisexual Identity 1996 (1(4) 295–310.

[37] Wehrum S, Klucken T, Kagerer S, Walter B, Hermann A, et al (2013) Gender Commonalities and Differences in the Neural Processing of Visual Sexual Stimuli. J Sex Med 10 (5) 1328–1342. 23421466 10.1111/jsm.12096

[38] Bradley MM, Lang PJ (1994) Measuring emotion: The self-assessment manikin and the semantic differential. Journal of Behavior Therapy and Experimental Psychiatry 25 (1) 49–59. 7962581 10.1016/0005-7916(94)90063-9

[39] Kirby KN, Gerlanc D (2013) BootES: An R package for bootstrap confidence intervals on effect sizes. Behav Res 45 (4) 905–927. 10.3758/s13428-013-0330-5 23519455

[40] R Core Team (2014) R: A language and environment for statistical computing. Available: http://www.R-project.org/.

[41] Kelley K (2005) The Effects of Nonnormal Distributions on Confidence Intervals Around the Standardized Mean Difference: Bootstrap and Parametric Confidence Intervals. Educational and Psychological Measurement 65 (1) 51–69.

[42] Shaffer JP (1986) Modified Sequentially Rejective Multiple Test Procedures. Journal of the American Statistical Association 81 (395) 826–831.

[43] Stippekohl B, Walter B, Winkler MH, Mucha RF, Pauli P, et al (2012) An early attentional bias to BEGIN-stimuli of the smoking ritual is accompanied with mesocorticolimbic deactivations in smokers. Psychopharmacology 222 (4) 593–607. 22476609 10.1007/s00213-012-2670-8

[44] Holm S (1979) A Simple Sequentially Rejective Multiple Test Procedure. Scandinavian Journal of Statistics 6 (2) 65–70 Available: http://www.jstor.org/stable/4615733.

[45] Rupp HA, Wallen K (2008) Sex Differences in Response to Visual Sexual Stimuli: A Review. Arch Sex Behav 37 (2) 206–218. 17668311 10.1007/s10508-007-9217-9 PMC2739403

[46] Laan E, Everaerd W, Bellen G, Hanewald G (1994) Women's sexual and emotional responses to male- and female-produced erotica. Arch Sex Behav 23 (2) 153–169. 7517135 10.1007/BF01542096

[47] Tsujimura A, Miyagawa Y, Takada S, Matsuoka Y, Takao T, et al (2009) Sex Differences in Visual Attention to Sexually Explicit Videos: A Preliminary Study. Journal of Sexual Medicine 6 (4) 1011–1017. 19175861 10.1111/j.1743-6109.2008.01031.x

[48] Brand M, Laier C, Pawlikowski M, Schächtle U, Schöler T, et al (2011) Watching Pornographic Pictures on the Internet: Role of Sexual Arousal Ratings and Psychological–Psychiatric Symptoms for Using Internet Sex Sites Excessively. Cyberpsychology, Behavior, and Social Networking 14 (6) 371–377. 10.1089/cyber.2010.0222 21117979

[49] Hogarth LC, Mogg K, Bradley BP, Duka T, Dickinson A (2003) Attentional orienting towards smoking-related stimuli. Behav Pharmacol 14 (2) 153–160. 12658076 10.1097/00008877-200303000-00007

[50] Bonnet M, Bradley MM, Lang PJ, REQUIN J (1995) Modulation of spinal reflexes: Arousal, pleasure, action. Psychophysiology 32 (4) 367–372. 7652113 10.1111/j.1469-8986.1995.tb01219.x

[51] Greenwald AG, McGhee DE, Schwartz JLK (1998) Measuring individual differences in implicit cognition: The implicit association test. Journal of Personality and Social Psychology 74 (6) 1464–1480. 9654756 10.1037//0022-3514.74.6.1464

[52] Hahn A, Judd CM, Hirsh HK, Blair IV (2014) Awareness of implicit attitudes. Journal of Experimental Psychology: General 143 (3) 1369–1392. 24294868 10.1037/a0035028 PMC4038711

[53] Posner MI, Rafal RD, Choate LS, Vaughan J (1985) Inhibition of return: Neural basis and function. Cognitive Neuropsychology 2 (3) 211–228.

